# Structural and Enzymatic Characterization of a Nucleoside Diphosphate Sugar Hydrolase from *Bdellovibrio bacteriovorus*

**DOI:** 10.1371/journal.pone.0141716

**Published:** 2015-11-02

**Authors:** Andres H. de la Peña, Allison Suarez, Krisna C. Duong-ly, Andrew J. Schoeffield, Mario A. Pizarro-Dupuy, Melissa Zarr, Silvia A. Pineiro, L. Mario Amzel, Sandra B. Gabelli

**Affiliations:** 1 Department of Biomedical Engineering, Johns Hopkins University School of Medicine, Baltimore, Maryland, United States of America; 2 Structural Enzymology and Thermodynamics Group, Department of Biophysics and Biophysical Chemistry, Johns Hopkins University School of Medicine, Baltimore, Maryland, United States of America; 3 Biology Department, Loyola University Maryland, Baltimore, Maryland, United States of America; 4 Department of Pharmacology and Molecular Sciences, Johns Hopkins University School of Medicine, Baltimore, Maryland, United States of America; 5 Department of Medical and Research Technology, University of Maryland School of Medicine, Baltimore, Maryland, United States of America; 6 Department of Medicine, Johns Hopkins University School of Medicine, Baltimore, Maryland, United States of America; 7 Department of Oncology, Johns Hopkins University School of Medicine, Baltimore, Maryland, United States of America; George Washington University, UNITED STATES

## Abstract

Given the broad range of substrates hydrolyzed by Nudix (*nu*cleoside *di*phosphate linked to *X*) enzymes, identification of sequence and structural elements that correctly predict a Nudix substrate or characterize a family is key to correctly annotate the myriad of Nudix enzymes. Here, we present the structure determination and characterization of Bd3179 –- a Nudix hydrolase from *Bdellovibrio bacteriovorus–*that we show localized in the periplasmic space of this obligate Gram-negative predator. We demonstrate that the enzyme is a nucleoside diphosphate sugar hydrolase (NDPSase) and has a high degree of sequence and structural similarity to a canonical ADP-ribose hydrolase and to a nucleoside diphosphate sugar hydrolase (1.4 and 1.3 Å Cα RMSD respectively). Examination of the structural elements conserved in both types of enzymes confirms that an aspartate-X-lysine motif on the C-terminal helix of the α-β-α NDPSase fold differentiates NDPSases from ADPRases.

## Introduction

*Bdellovibrio bacteriovorus* is a highly motile obligate predatory bacterium. It employs a large repertoire of hydrolases (the second largest density of hydrolases per genome [1, 2]) to prey and devour other Gram-negative bacteria. There are no reports of mammalian cells being targeted by *Bdellovibrio bacteriovorus*, and its lack of type III and IV secretion systems [2] makes it an ideal candidate for use as a live antimicrobial agent in humans. Furthermore, the large number of hydrolases and the precise timing of expression in its intricate biphasic life cycle can illuminate novel enzyme-based antimicrobial mechanisms and drug targets. Of particular interest is the *Bdellovibrio bacteriovorus* HD100 nucleoside diphosphate sugar (NDPS) hydrolase Bd3179 [3] because it appears to have some of the characteristics of the *Escherichia coli* adenosine diphosphate ribose (ADPR) hydrolase [4, 5] and the guanosine diphosphate mannose (GDPM) hydrolase [6]. A structural comparison of these three Nudix (*nu*cleoside *di*phosphate linked to *X*) hydrolases may provide clues about the structural determinants of substrate specificity of the Nudix superfamily.

Nudix enzymes have been identified in over 20,000 species [7] representing all kingdoms of life by using the signature amino acid sequence $G_{1}^{N}[5X]E_{7}^{N}[7X]R_{15}^{N}E_{16}^{N}XXE_{19}^{N}E_{20}^{N}XG_{22}^{N}U$ (where U is I, L or V and superscript N denotes that the numbering refer to a residue of the signature sequence). The ubiquitous nature of Nudix enzymes arises from the versatility of the Nudix fold which has been evolutionarily repurposed to hydrolyze a plurality of substrates as in the ADPRase, A4pAase, CoAase, mRNA decapping, and antimutator families [1, 5, 8–16]. The signature motif is located in a loop-helix-loop substructure within the Nudix fold. While the signature motif is highly conserved, the Nudix fold can accommodate extensions in its β-strands and their connecting loops. These variations and additional domains confer the enzymes the ability to recognize a plurality of substrates, but make it difficult to identify sequence elements that are unique to the different families of Nudix hydrolases. In cases where structural information is available, as in the case of the well-studied ADPRases, sequence elements that correctly predict Nudix substrates have been identified. For example, a proline 15 amino acids downstream of $G_{22}^{N}$ has been used to correctly predict substrate preference for ADPR [17]. *E*. *coli* GDPMase exhibits the same fold and $G_{22}^{N}$+15 pattern as the ADPRases, yet preferentially hydrolyzes two other nucleoside diphosphate sugars over ADPR [6], demonstrating that other structural elements must be at play to differentiate the sugar derivative substrates. In this paper we describe the cellular localization, the determination of the structure and the *in vitro* substrate specificity of Bd3179.

In light of findings described herein, we propose to rename the GDPMase Nudix family as an NDPSase Nudix family, since these enzymes hydrolyze at least three other NDPS analogues in addition to GDPM. The function of this family remains unknown. In *E*. *coli*, it was believed to regulate biofilm formation since it forms part of the RcsC regulon [18], but knockout studies did not show a significant effect on biofilm formation [6]. The ADPRase family has been extensively studied and is believed that, among other functions, its members regulate the ADPR pool and prevent deleterious non-enzymatic ribosylation [5, 17, 19]. Despite these distinct functions, both Nudix families share a high degree of structural homology that can be used to guide sequence-based identification of their substrate specificity. By comparing the sequence elements to the substrate bound structures of *Escherichia coli* ADPRase [4] and NDPSase [6] we propose that an aspartate-X-lysine sequence motif on the C-terminal helix of the Nudix fold differentiates NDPSases from ADPRases.

## Materials and Methods

### Cloning and site directed mutagenesis of *Bdellovibrio bacteriovorus Bd3179*

The *Bd3179* gene was amplified from *Bdellovibrio bacteriovorus* HD100 chromosomal DNA by PCR and cloned into the expression vector pET24a (Novagen, Madison WI). The E140Q site directed mutation was introduced using the QuikChange Kit (Stratagene).

### Bd-NDPSase protein expression and purification

BL21 (DE3) cells were transformed with a pET24a (Novagen, Madison WI) plasmid containing the *Bdellovibrio bacteriovorus Bd3179* gene. Cells were grown at 37°C from a single colony inoculum in LB media supplemented with Kanamycin. Bd-NDPSase expression was induced at an OD_600_ of 0.7 with 100 μM IPTG. Cells were grown for three to five hours, centrifuged, pelleted, and frozen at -80°C.

The cell pellet was thawed, resuspended in 80 ml TE buffer (50 mM Tris-HCl pH 7.5, 1 mM EDTA), and lysed with a microfluidizer 2x at 75 psi. The cell lysate was clarified by centrifugation for 30 minutes at 11,500 rpm using TE buffer at 0, 30, and 60% (w/v) ammonium sulfate concentrations. The pellet from the last clarification step was resuspended in TE buffer at a 60% (w/v) ammonium sulfate concentration, filtered with a Corning PES 0.20 μm filter, and loaded on a hydrophobic interaction column (Phenyl FF HiPrep 16/10, GE Healthcare) equilibrated in resuspension buffer. Bound protein was eluted with a TE buffer gradient. Fractions containing protein were dialyzed at 4°C 2x against 2 L of 50 mM Tris-HCl pH 8.6, 1 mM EDTA buffer overnight in 3500 Da dialysis tubing (SnakeSkin, Thermo Scientific), and loaded onto an anion exchange column (Resource Q, GE Healthcare). The protein was loaded with 50 mM Tris-HCL pH 8.6, 1 mM EDTA (Buffer A) and eluted with a 50 mM Tris-HCl pH 8.5, 1 mM EDTA and 1 M NaCl (Buffer B) gradient. Combined eluted fractions were concentrated and the purity of the sample (>90%) was assessed by SDS-PAGE.

### Protein crystallization

Wild type and E140Q Bd-NDPSase crystals were grown by hanging drop vapor diffusion at 20°C. The drop, containing 1 μl of the reservoir solution (1.75–2.0 M ammonium sulfate, 0.1 M HEPES pH 7.0, and 0–0.5% PEG 8000) and 1 μl of 9.5–11.0 mg/ml of enzyme in 50 mM Tris-HCl pH 8.6, 1 mM EDTA buffer was equilibrated over 1 mL of reservoir solution. Crystals were derivatized with 5mM SmCl_3_ for 48 hours, and either UO_2_(ClO_4_)_2_ or 1 mM GdCl_3_ for 5 days in preparation for multiple isomorphous replacement. Crystals of the Bd-NDPSase-ADPR binary complex were obtained by soaking crystals of the E140Q mutant in 5 mM ADPR for 2 days. Co-crystallization of the Bd-NDPSase with 5 mM UDPG did not give the expected complex. Instead, the crystals contained the Bd-NDPSase-glucose complex.

### Diffraction data collection, structure determination, and refinement

Data for the native crystal were collected at beam line X6A of the Brookhaven National Laboratory, National Synchrotron Light Source. All heavy atom derivative data were collected on a copper rotating anode x-ray generator (RU-H3R) as the X-ray source and an RAXIS IV (Rigaku) as the detector, at the X-ray facility of the Department of Biophysics and Biophysical Chemistry of the Johns Hopkins University School of Medicine. Indexing and data reduction were carried out with the HKL2000 suite [20]. Phases were calculated using the SmCl_3_, UO_2_(CLO_4_)_2_ and GdCl_3_ derivatives with the program SOLVE [21, 22]. SmCl_3_ and GdCl_3_ had two sites each and UO_2_(CLO_4_)_2_ had three sites with a combined phase figure of merit of 0.49 to 2.89 Å. Density modification and an automatic partial model were built using the program RESOLVE [23, 24]. The final model was built and refined using iterative cycles of manual building and maximum likelihood refinement with the programs Coot and REFMAC5 of the CCP4 suite [25–28]. Data statistics are shown in Table 1.

**Table 1:** Data collection and refinement statistics for Bd-NDPSase.

	Wild typeGlycerol	E140Q Glucose	E140Q ADPR	Wild type SmCl_3_	Wild type UO_2_(ClO_4_)_2_	Wild type GdCl_3_
**Data collection**						
Space group	P2_1_2_1_2	P2_1_2_1_2	P2_1_2_1_2	P2_1_2_1_2	P2_1_2_1_2	P2_1_2_1_2
Cell dimensions						
*a*, *b*, *c* (Å)	75.4, 103.1,51.7	75.9, 103.3, 51.7	75.6, 103.4, 51.6	75.8, 103.4, 51.8	76.3, 103.0, 51.7	76.1, 103.5, 52.0
α, β, γ (°)	90, 90, 90	90, 90, 90	90, 90, 90	90, 90, 90	90, 90, 90	90, 90, 90
Resolution (Å)	50.00–1.52 (1.57–1.52)	50.00–1.80 (1.86–1.80)	50.00–2.06 (2.13–2.06)	50.00–2.43 (2.52–2.43)	50.00–2.52 (2.43–2.52)	50.00–1.95 (2.02–1.95)
Wavelength (Å)	0.948	1.542	1.542	1.542	1.542	1.542
R_symm_ (%)	11.1 (63.6)	6.4 (46.6)	9.5 (52.5)	11.6 (45.9)	11.5 (41.6)	7.7 (48.0)
I/Sigma	23.9 (1.38)	42.8 (3.04)	17.6 (2.3)	23.6 (3.5)	27.8 (4.6)	35.7 (2.5)
Completeness (%)	94.5 (80.9)	92.4 (54.1)	95.0 (68.9)	99.9 (99.9)	95.4 (95.1)	90.8 (84.9)
Unique Reflections	58,801	35,689	24,431	15,953	15,329	27,794
Total Reflections	366,440	201,670	130,858	94,097	89,552	159,751
Redundancy	6.2 (3.9)	5.7 (3.4)	5.4 (2.1)	5.9	5.8	5.7
**Refinement**						
R_work_/R_free_ (%)	0.18/0.21	0.16/0.20	0.18/0.24			
No. atoms						
Protein	3,014	3,016	2,984			
Ligand	134	85	169			
H_2_O	349	320	212			
B-factors						
Protein	21.70	27.74	29.70			
Water	33.17	36.99	37.10			
Ligand	39.70	52.10	61.29			
RMS deviations						
Bond lengths (Å)	0.013	0.012	0.015			
Bond angles (°)	1.641	1.497	1.886			

Numbers in parenthesis show the values for the highest resolution shell

The ligand-bound structures were built in a Fourier map using phases of the wild type protein. The initial model was rebuilt in Coot [27] and refined using REFMAC5 [29]. Quality of the structures was assessed with Coot [27]. Figures were drawn with PyMOL [30].

### Substrate determination assay

A panel consisting of nucleotides and nucleoside diphosphate derivatives, assayed for hydrolysis by Bd-NDPSase, revealed a preference for nucleoside diphosphate sugars. Based on its molecular weight, dimer quaternary arrangement and sequence homology to Nudix hydrolases such as the Ec-ADPRase and Ec-NDPSase, a panel consisting primarily of nucleoside diphosphate sugars was assayed to determine the highest relative activity of Bd-NDPSase. These substrates included GDP-mannose (GDPM), GDP-glucose (GDPG), UDP-glucose (UDPG), and ADP-ribose (ADPR). The colorimetric assay measures phosphate release as described by Ames [31]. Assays were carried out in 50 μl volumes with 2 mM of substrate, 200 nM of enzyme (wild type or E140Q Bd-NDPSase), 20 units/ml of calf intestinal phosphatase (NEB), and 5 mM MgCl_2_ at 37°C for 15 minutes. The reaction was quenched with 250 μl of 30 mM EDTA, and developed with 700 μl of Ames solution (0.42% ammonium molybdate in 1 N H_2_SO_4_) at 42°C for 20 minutes. Relative activity was determined by measuring absorbance at A_820_ and normalizing by the highest reading.

### Kinetics assay

The phosphate release assay was used to determine the kinetics of Bd-NDPSase and E140Q Bd-NDPSase using GDP-mannose as the substrate. 50 μl of a 350 μl reaction solution consisting of GDP-mannose (0, 0.2, 0.4, 0.6, 0.8, and 1 mM), 50 mM Tris-HCl pH 8.6, 1 mM EDTA buffer, 5 mM MgCl_2_, 2.5 mM enzyme, and calf intestinal phosphatase (NEB) were taken every 2 minutes. The reaction for each time point was quenched with 30 μl of 100 mM EDTA and placed on ice. 220 μl of ddH_2_O was added to each time point, followed by the addition of 700 μl of Ames solution. These were developed at 42°C for 20 minutes. The concentration of the phosphate liberated was measured at A_820_. A standard curve was used to calculate the amount of hydrolyzed substrate. Initial rates of GDPM hydrolysis were fit by nonlinear least squares to the Michaelis-Menten equation to determine k_cat_ and K_m_.

### Preparation of rat anti-Bd-NDPSase serum

Rat antisera were developed using a modification of the procedure by Larsson and Nilsson [32]. Ten μL of Bd-NDPSase at 9.3 mg/mL was placed onto each of two one-cm^2^ coupons of sterile nitrocellulose paper and allowed to air dry. Each nitrocellulose coupon was then surgically implanted into the peritoneal cavity of a Sprague Dawley rat. The procedure was repeated on days 10, 20 and 30 with similarly prepared coupons. On day 40, rats were exsanguinated via heart puncture. The resulting sera was stored at -20°C.

### Immunoelectron microscopy of Bd-NDPSase

A 100 mL *Bdellovibrio bacteriovorus* HD100 liquid culture co-incubated for 7 days with *E*. *coli* prey at 30°C was filtered sequentially with 0.80 μm and 0.45 μm filters as described before [33]. The filtrate was centrifuged at 20,000 g for 30 minutes. The resulting free-living attack phase *Bdellovibrio bacteriovorus* pellets were fixed in 4% formaldehyde in 0.1 M sodium cacodylate buffer with 3% sucrose and 3 mM CaCl_2_ [34]. Fixed bacteria were then cryopreserved in 2.3 M sucrose in 20% polyvinylpyrrolidone (Sigma). The bacteria were then frozen in liquid nitrogen. Ultra-thin sections were cut with a Leica UCT microtome and the sections were placed on 200 mesh nickel formvar coated grids. Grids were floated section side down on drops of primary antibody diluted in PBS with 10% fetal bovine serum overnight at 4°C. Primary antibody was detected with 12 nM goat anti-rat secondary gold antibody (Jackson ImmunoResearch, Inc. West Grove, Pennsylvania) diluted 1:20 in PBS for 1 hour at room temperature. Final contrasting of the sections was done by incubating them in 2% methyl cellulose (Sigma) and 0.3% uranyl acetate (Ted Pella) for 10 minutes at 4°C. All sections were viewed on a Philips CM 120 TEM at 80 kV, equipped with a Gatan Orius SC 1000 digital camera.

### Structural and sequence alignment

Structural alignment of Bd-NDPSase to other Nudix enzymes was performed using PDBeFold [35]. The structural homologues (PDB IDs 3O61 and 1KHZ) with the lowest Cα RMSD [35] (1.3 and 1.4 Å) containing metal and substrate in the active site were compared to Bd-NDPSase using PyMOL [30] and LigPlot [36]. The ENDscript [37] web server was used to display and analyze the alignment (S1 Fig). Cavity and dimer interface surface areas were computed using PISA [38]. The amino acid sequences of the structural homologues (PBD IDs 3O61 and 1KHZ) were aligned to Bd-NDPSase using ClustalW [39] and visualized using ESPript [40]. The structural alignment produced by PDBeFold was used to manually improve the sequence alignment on strand β3 [35].

### Protein structure accession numbers

Atomic coordinates and structure factors of the wild type Bd-NDPSase in complex with glycerol (PDB ID 5C7Q), E140Q Bd-NDPSase in complex with ADP-ribose (PDB ID 5C7T), and E140Q Bd-NDPSase in complex with glucose (PDB ID 5C8L) were deposited in the Protein Data Bank.

## Results and Discussion

### Cellular localization of *Bdellovibrio bacteriovorus* NDPSase

**Fig 1:**
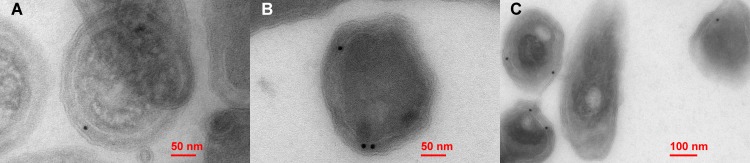
Cellular localization of Bd-NDPSase. **A-B)** TEM micrographs at 37,000x magnification showing the location of the secondary gold antibody (black dots) in the periplasmic space of a *Bdellovibrio bacteriovorus* cell. **C)** TEM micrographs at 20,000x magnification showing the location of the secondary gold antibody (solid, black spots) in the periplasmic space of multiple *Bdellovibrio bacteriovorus* cells.

We determined the subcellular localization of Bd-NDPSase by raising a polyclonal antibody against the purified enzyme. The primary antibody, visualized by transmission electron microscopy (TEM) using a gold particle-conjugated secondary antibody, shows that Bd-NDPSase is localized in the periplasm of the cells (Fig 1). We suggest that, in addition to structural determinants, the concentrations of Nudix substrates in the periplasm of Bd-NDPSase may be an important determinant of its physiological substrate preference.

Previous studies, listing the expression profiles of host dependent (attack and 30 minutes post-invasion phase) and host independent *Bdellovibrio bacteriovorus* strains, have shown that *Bd3179* gene is not included in the group of up or down regulated genes [41, 42]. Thus we speculate that Bd-NDPSase periplasmic location might be associated with metabolizing host material in the bdelloplast after the 30 minutes initial invasion of the prey cell. Moreover, the studies from the Sorek laboratory showing expression of Bd3179 at the growth phase also suggests that Bd3179 could have a role when *Bdellovibrio* degrades and metabolizes host cell [43].

### Bd-NDPSase *in vitro* substrate

Identification of a Nudix enzyme endogenous substrate has proved elusive largely due to the tendency of Nudix enzymes to hydrolyze more than one substrate *in vitro*. For instance, MutX hydrolyses all 8 canonical nucleotide triphosphates [44]. Furthermore, substrate specificity is often a function of the divalent cation used for *in vitro* studies. In the absence of a *de facto* standard to determine the metal cofactor *in vivo*, most Nudix studies have been conducted assuming that magnesium or manganese [14, 45–49] is the endogenous cofactor and determining the best substrate based on their K_m_ and k_cat_ or their specificity constant k_cat_/ K_m_. However, functional knockout studies have shown that in some cases the preferred *in vitro* substrate is not necessarily the physiological substrate. For example, *Arabidopsis thaliana nudt7 [50]* and *Mycobacterium tuberculosis renU [51]* functional knockouts did not show an effect on the *in vivo* concentration of the best *in vitro* substrate, ADPR. Instead, these knockouts showed an accumulation of NADH [50, 51], which, for the mycobacterial enzyme, showed a 10-fold lower specificity constant than ADPR [51].

**Fig 2:**
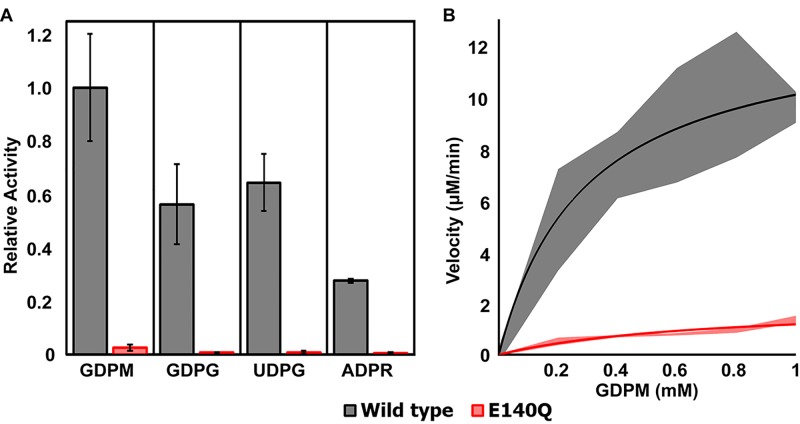
Bd-NDPSase wild type and E140Q substrate specificity. **A)** Wild type enzyme (gray bars) exhibited preference for nucleoside diphosphate sugar (NDPS). E140Q mutant (red bars) were catalytically inactive. **B)** Initial rates of GDPM hydrolysis for the wild type and E140Q mutant were fit by nonlinear least squares to the Michaelis-Menten equation (solid lines) to determine k_cat_ (5.2 (ms)^-1^) and K_m_ (0.3 mM). Standard deviations of triplicate measurements are shown by the shaded bars for the wild type (gray shade) and mutant (red shade).

Phosphate release assays revealed that among the nucleoside diphosphate sugars hydrolyzed by Bd-NDPSase, its highest relative activity was with GDPM. UDPG, GDPG, and ADPR, were also hydrolyzed, but at a lower extent than GDPM (Fig 2A). Michaelis-Menten kinetics revealed a k_cat_ of 5200 s^-1^ and K_m_ of 0.3 mM for GDPM. This K_m_ is comparable to those of other Nudix sugar hydrolases with a similar structure and molecular weight [5, 6, 17, 19, 52, 53]. Given its cellular localization it is likely that the physiological substrate of Bd-NDPSase will be determined by which compounds are present at high concentration in the periplasmic space or become available from the host cell when *Bdellovibrio* degrades it.

### Overall structure

**Fig 3:**
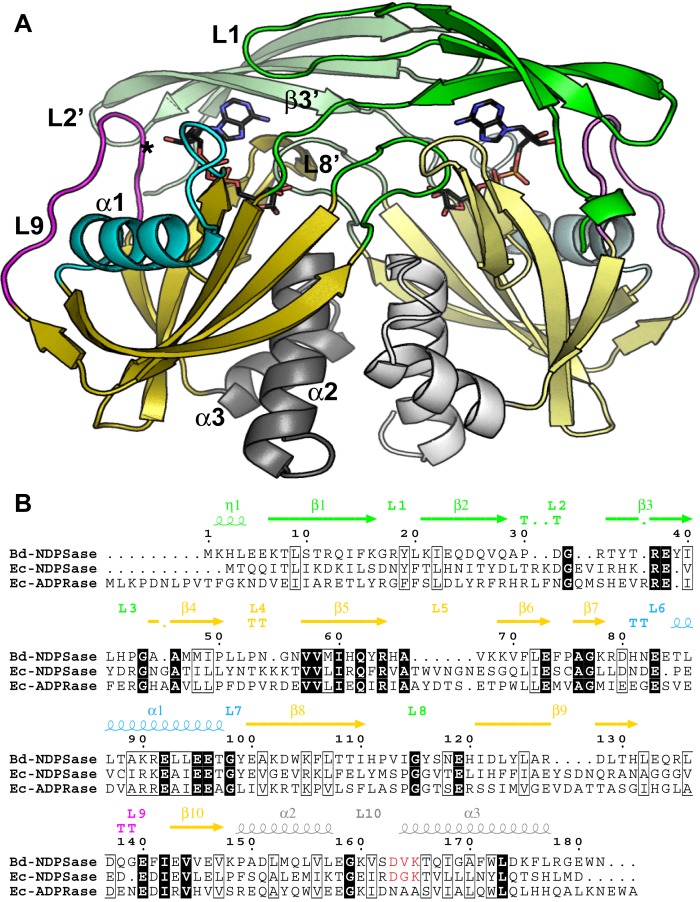
Overall Structure Bd-NDPSase. **A)** Structure of E140Q Bd-NDPSase bound to ADPR (PDB ID 5C7T). **B)** Sequence alignment of the Ec-ADPRase (PDB ID 1KHZ) and Ec-NDPSase (PDB ID 3O61) to Bd-NDPSase. The N-terminal domain (residues 1–44) consists of an antiparallel beta sheet (β1- β3) and is denoted by green. The Nudix fold consists of a mixed beta sheet (β1- β10, yellow) flanked by helix α1 (cyan) on one side and helices α2 and α3 (residues 148–182, gray) on the other side. The location of the E140Q mutation on loop L9 (magenta) is denoted by an asterisk. The prime symbol (‘) denotes residues of the opposite monomer (lighter color shade).

Bd-NDPSase is a homodimer whose monomers are related by a non-crystallographic 2-fold axis. Each monomer comprises an N-terminal and a Nudix domain (Fig 3). The N-terminal domain contains a beta sheet composed of three anti-parallel strands (residues 1–40) connected by loops. Loop L1, which connects strands β1 and β2, contributes to a π-stacking interaction with the substrate *via* Y19. The C-terminal domain, comprising the Nudix fold, is composed of an α-β-α motif (α1, β4-β10, α2). The specificity loop L8 joining strands β8 and β9 of the mixed beta sheet interacts with the substrate in the catalytic site of the opposite monomer via hydrogen bonding. The swapped N-terminal domains and the Nudix fold form two catalytic cavities with an exposed surface of ~689 Å^2^ each. The signature Nudix sequence is folded as loop-helix-loop (β7-L6-α1-L7). Its conserved glutamates $E_{16}^{N},E_{19}^{N}{,E}_{20}^{N}$, together with E140 of loop L9, are positioned to coordinate Mg^2+^ binding.

An extensive dimer interface (2,992 Å^2^) spans the largest axial cross-section of the homodimer. The dimer interface spans both N-terminal and C-terminal domains and separates the Nudix folds of each monomer along the non-crystallography 2-fold axis. It includes interactions between homologous domain elements as well as domain swapped elements (S2 Fig). Not surprisingly, substrate binding involves elements from both monomers. In fact, 46% of the buried ADPR surface in each active site is from the opposite monomer.

### ADPR recognition

**Fig 4:**
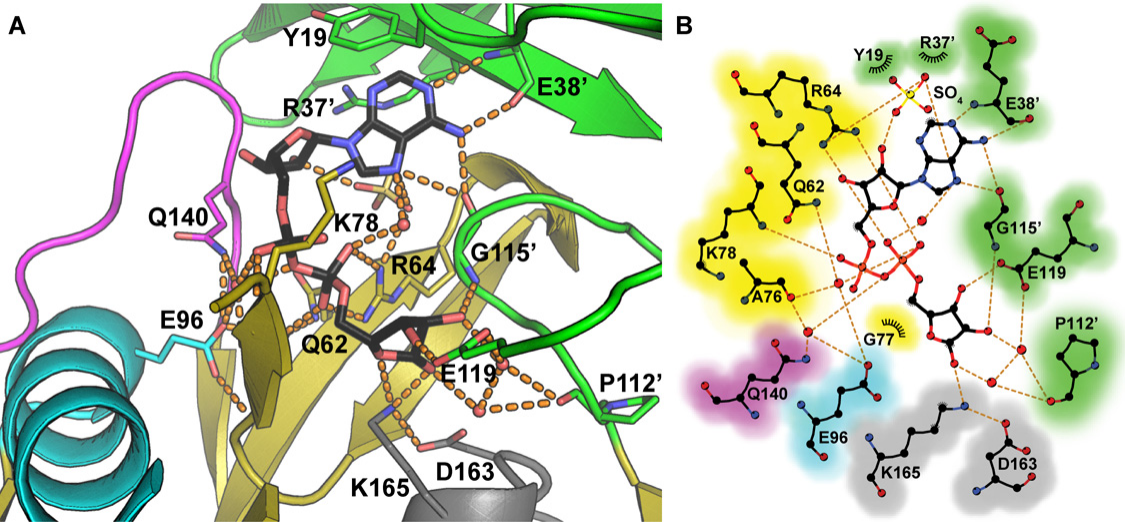
Recognition of ADPR by Bd-NDPSase. **A)** Ribbon representation of the catalytic site of the ADPR-bound E140Q Bd-NDPSase crystal structure (PDB ID 5C7T). **B)** Schematic representation of the recognition of ADPR by Bd-NDPSase. Catalytic helix (α1) residues are shown in cyan, catalytic loop L9 residues are shown in magenta. N-terminal domain residues (1–44) are shown in green as is the specificity loop L7. Hydrogen bonds are shown as orange dashes. The prime symbol (‘) denotes residues of the opposite monomer.

**Fig 5:**
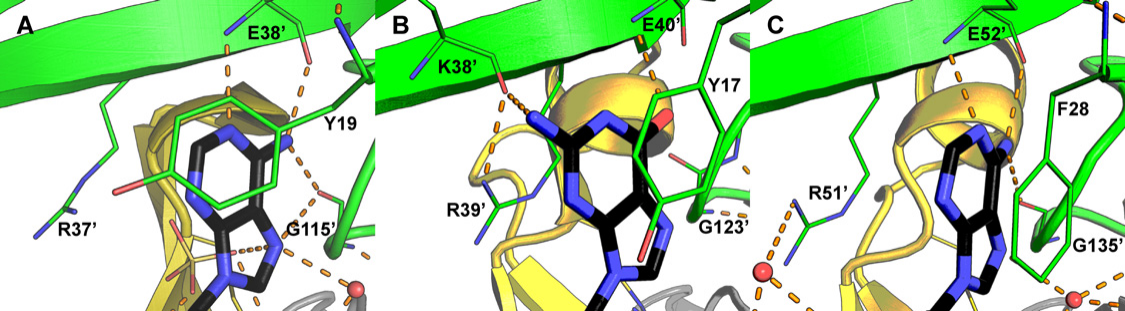
Nucleoside recognition by bacterial Nudix sugar hydrolases. In all cases, the nucleoside is stacked by an aromatic residues in loop L1 and an arginine in strand β3’. **A)** Adenosine recognition by the Bd-NDPSase hydrolase (PDB ID 5C7T). **B)** Guanosine recognition by the Ec-NDPSase hydrolase (PDB ID 3O61). **C)** Adenosine recognition by the Ec-ADPR hydrolase (PDB ID 1KHZ). Substrate carbons are shown in black, residue carbons are colored using the main chain color convention. Nitrogen and Oxygen are colored in blue and red respectively. Hydrogen bonds are shown as orange dashes. The prime symbol (‘) denotes elements of the opposite monomer.

Adenosine recognition involves hydrogen bonding by the E38’ (prime denotes a residue of the other monomer) main chain amide nitrogen and carbonyl oxygen to the adenosyl N1 and N6 respectively. The main chain oxygen of G115’ bridges the adenosyl N6 and N7. R37’ and Y19 flank the nucleoside base though stacking interactions on opposite sides (Figs 4 and 5A). Analogous stacking interactions, involving the absolutely conserved arginine on strand β3 and the conserved aromatic residue on loop L1 are also present in Ec-NDPSase and Ec-ADPRase (Fig 5).

**Fig 6:**
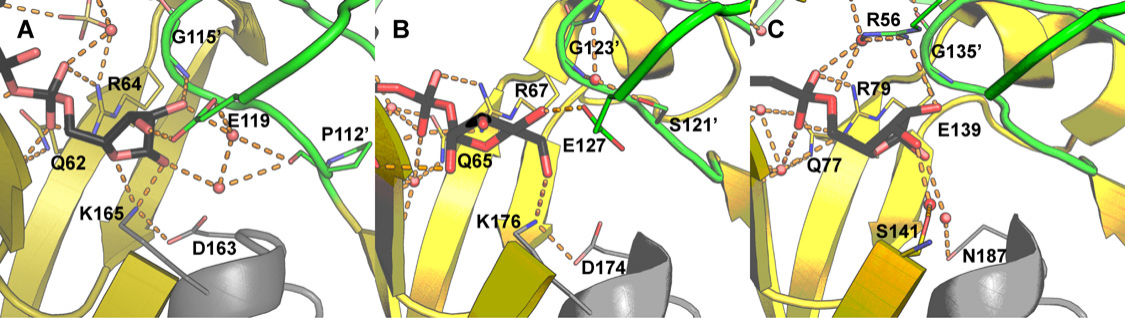
Sugar recognition by bacterial Nudix sugar hydrolases. **A)** Ribose recognition by the Bd-NDPSase (PDB ID 5C7T). **B)** Mannose recognition by the Ec-NDPS hydrolase (PDB ID 3O61). **C)** Ribose recognition by the Ec-ADPR hydrolase (PDB ID 1KHZ). Substrate carbons are shown in black, residue carbons are colored using the main chain color convention. Nitrogen and Oxygen are colored in blue and red respectively. Hydrogen bonds are shown as orange dashes. The prime symbol (‘) denotes residues of the opposite monomer.

As in Ec-NDPSase [6], Ec-ADPRase [4], and Mt-ADPRase [5], R64 of Bd-NDPSase forms a bidentate hydrogen bond through the guanidinium side chain with the β-phosphate oxygens O1β and O2β (Fig 6). It should be noted that this mode of binding is present in metal-substrate bound ternary structures as well as substrate bound binary structures [4, 6]. This suggests that, in addition to serving as a Lewis acid to facilitate release of ribose phosphate [54], R64 recognizes and orients the β-phosphate of the substrate. Mutational studies in a homologous Nudix hydrolase suggest that R64 is involved in both substrate binding and catalysis as evidenced by an increase in K_m_ and a decrease in k_cat_ upon arginine substitution by glutamate [55]. Comparison of the substrate bound Ec-NDPSase to the Ec-ADPRase reveals that the highly conserved arginine on strand β3 (Bd-NDPSase R64) is ideally positioned for charge compensation of the sugar-phosphate product (Figs 4 and 6A).

In addition to nucleoside and phosphate recognition, Bd-NDPSase forms a network of hydrogen bonding interactions involving the three hydroxyl groups of the distal ribose. G115’ tethers both ends of an ADPR horseshoe conformation by hydrogen bonding to the hydroxyl at ribose C2 through its main chain amide nitrogen as well as to adenosine through the amide oxygen (Fig 4). The carboxylate of E119 makes a hydrogen bond with the hydroxyl at C3 and positions a water molecule to make a hydrogen bond to the hydroxyl on C2 (Fig 6). The most distinguishing amino acid residues are K165 and D163, which form a bridge between the hydroxyl at C1 and the hydroxyl at the Cβ of T166. Interestingly, this network of interactions is also present in Ec-NDPSase, but not in Ec-ADPRase, suggesting that these residues may indeed be responsible for differentiation between GDPM and ADPR [6].

The proposition that E140 functions in catalysis is strengthened by the fact that substitution by glutamine renders the enzyme catalytically inactive, as evidenced by both relative activity assays and kinetic assays (Fig 2). The B-factor of the E140 Cα in apo Bd-NDPSase is 3.1 times larger than that of G115’ on loop L8, and 1.7 times larger than that of Y19 on loop L1, suggesting that loop L9 intrinsic flexibility facilitates substrate binding followed by E140 mediated nucleophilic attack of the scissile phosphate. Another plausible mechanism employing L9 displacement is that proposed by Nakamura [56] in which a third cation neutralizes the negative charge buildup of the transition state concomitantly with the displacement of an aspartate residue.

Comparison of the ADPR-bound Bd-NDPSase with the apo structure (PDB ID 5C7Q) does not readily reveal a product-release mechanism since active site residues are not displaced upon substrate binding and there is no significant change in tertiary structure (Fig 5 and S3 Fig). A product-release mechanism where an arginine (R37 in Bd-NDPSase) completes a “fence” around the nucleoside [53] is posible, but the presence of a sulfate ion in close proximity (4.5 Å) of R37 in the apo structure precludes this analysis. A product-release mechanism where a glutamate on loop L9 (E140 in Bd-NDPSase) gates the substrate in the Mg^2+^-ADPR ternary complex [6] is also plausible.

### Glucose recognition

Bd-NDPSase crystals soaked with UDPG displayed electron density that could be interpreted as a glucose molecule in each active site of the asymmetric unit. Modeling of glucose, sulfate, and glycerol molecules in the unbiased electron density revealed that these molecules are located within hydrogen bonding distance of catalytic residues (S4 Fig). R64 makes a hydrogen bond with the sulfate and glucose molecules through its guanidinium side chain. Water molecules mediate the interactions of these molecules with other key residues such as E96, Q140, and K165. The glucose molecule is shifted toward the expected position of the phosphate of a phosphohexose product (S4 Fig).

### Modularity of Bd-NDPSase

The phylogenetically conserved Nudix enzymes appear to have been tuned to perform one catalytic activity, the hydrolysis of a diphosphate or a phosphate ester, and evolutionarily repurposed to perform diverse functions in the cell. Their versatility can be correlated with their modular design where substrate recognition is independent from substrate hydrolysis. In fact, several Nudix proteins have been characterized as proteins that bind, but do not hydrolyze substrates [57–59]. An analysis of the structure of the dimeric Bd-NDPSase in light of other available Nudix enzyme structures, suggests subdivision of each monomer into three regions with exclusive roles: 1) the Nudix fold (Fig 3: magenta, cyan, yellow) composed of the α-β-α motif (α1, β4-β10, α2); 2) the nucleoside recognition module (Fig 3: green) composed of L1, L8’ and β3’ of the N-terminal domain; and 3) the sugar recognition module (Fig 3: green, gray) composed of L8, L8’ and α3 of the Nudix fold. In turn, the Nudix fold can be subdivided into three modules with different functions: a *metal binding* module formed by the glutamates of the Nudix signature sequence (Fig 3: cyan); a *phosphate charge compensation* module formed by amino acids in β5 (Fig 3: yellow); and a *catalytic base gating* module formed by loop L9 ($G_{22}^{N}$+ ~40; Fig 3: magenta). Changes in each of these modules repurpose the enzyme for different substrates and consequently for a specific physiological function. Importantly, they could also be used to target these enzymes in the laboratory as a designed experimental tool.

### Differentiation of ADPRases and NDPSases

Despite the low degree of sequence identity and similarity (13.7% and 16.4% respectively) between the Ec-ADPRase, Ec-NDPSase and Bd-NDPSase there is a high degree of similarity in their structures as well as in their quaternary arrangement, as shown by low RMSD 1.34 and 1.4 Å respectively for 255 Cα aligned (S5 Fig). Sequence identity and similarity are accounted for mostly by the Nudix fold. The N-terminal domains contain four identical residues and four similar residues. Of these residues, only Y19, R37’, and E38’mediate nucleoside recognition (Fig 5A). It is worthwhile noting that the mechanism for nucleoside recognition is conserved across all three proteins. The hydrophobic residue in loop L1 stacks against the nucleoside base while the tandem arginine and glutamate of strand β3 mediate adenosine or guanosine recognition (Fig 5). The mechanism for recognition of the β-phosphate is likewise conserved in all three homologues, with the arginine at the end of strand β5 forming hydrogen bonds through its guanidinium side chain to the β-phosphate oxygens O1β and O2β. Therefore, differentiation between the substrates ADPR and GDPM must take place through a mechanism involving the derivative ribose and mannose as suggested by Boto [6]. The present work supports this hypothesis since the same hydrogen bond network, involving the aspartate and lysine at the N-terminus of helix α3, is conserved only in Bd-NDPSase (PDB ID 5C7T, this work) and Ec-NDPSase (PDB ID 3O61[6]).

ADPRases have been previously identified through sequence elements alone. The ADPRases in *Haemophilus influenza* and *Bacillus subtilis* were correctly identified by Dunn [17] using a proline 15 amino acids downstream from the C-terminal glycine of the signature Nudix motif ($G_{22}^{N}$+15). Likewise, Boto [6] hypothesized that an aspartate-X-lysine motif at the N-terminus of helix α3 in Ec-NDPSase would correctly predict substrate preference for GDPM over ADPR. This aspartate-X-lysine pair ($G_{22}^{N}$+65), in fact, correctly predicted that Bd-NDPSase prefers GDPM over ADPR. Furthermore, the highly conserved Proline ($G_{22}^{N}$+15) is not a necessary condition for NDPSase characterization as Bd-NDPSase possesses an isoleucine at this position. Both Ec-NDPSase and Bd-NDPSases also hydrolyzed UDPG preferentially over ADPR. We thus propose that this type of enzymes, characterized by a domain swapped N-terminal dimer, a signature Nudix motif in each molecule, and an aspartate-X-lysine motif on helix α3 be known as nucleoside diphosphate sugar hydrolase, “NDPSase.”

While the Nudix fold is largely responsible for recognition of the derivative sugar and diphosphate, the N-terminal domain recognizes the nucleoside base. The highly conserved E38’ of Bd-NDPSase β3 forms a hydrogen bond with adenosine N5 and N6 through the main chain amide nitrogen and oxygen, respectively (Fig 5A and 5C). As evidenced by the GDPM bound structure of Ec-NDPSase, for guanosine recognition (Fig 5B) to take place, it needs only to be displaced ~2.5 Å from the adenosine position (Fig 5A and 5C). This displacement positions the guanosine within distance to form a hydrogen bond with the main chain amide nitrogen of the highly conserved glutamate in β3 and the main chain amide oxygen of the residue two positions upstream (Fig 5B). This mechanism explains how the NDPSases can recognize multiple nucleosides and, with the addition of the aspartate-X-lysine motif at α3, differentiate between the mannose and ribose derivative sugars.

## Supporting Information

S1 Fig Structurally guided sequence alignment. Chain A of the PDB entries 1MK1, 1V8I, 2YVM, 1VHZ, 3ACA, 2DSC, 3BM4, 1GOS, and 3O61 were aligned to chain A of Bd-NDPSase using the ENDscript web server. Accessibility: white is buried (A < 0.1), cyan is intermediate (0.1 ≤ A ≤ 0.4), blue is accessible (0.4 < A ≤ 1). Hydropathy: pink is hydrophobic (H>1.5), gray is intermediate (-1.5 ≤ H ≤ 1.5), and cyan is hydrophilic (H < -1.5). Contacts: yellow background is a non-crystallographic contact, orange background is both a crystallographic and a non-crystallographic contact, a red letter is a short range contact (ℓ < 3.2 Å), a black letter is a long range contact (3.2 Å ≤ ℓ ≤ 5.0 Å), an asterisk is a ligand contact, A and B denote the interacting chain.
(TIF)

S2 Fig Bd-NDPSase dimer interface. The dimer interface was calculated using the web server PISA. Structural elements are colored as in Figs 2–5. The side chains involved in dimer contacts are represented by red ribbons (**A** and **C**) and a red surface (**B** and **D**). Panels **C-D** are viewed at a 90° rotation from **A-B**.
(TIF)

S3 Fig Active site residues in ADPR-bound and apo Bd-NDPSase. Ribbon representation in which wild type Bd-NDPSase bound to glycerol (PDB ID_5C7Q) is shown in gray and E140Q Bd-NDPSase bound to ADPR (PDB ID 5C7T) is shown in blue. One chain of the dimer is shown in a lighter shade. Substrate carbons are shown in black, residue carbons are colored using the main chain color convention. Nitrogen and oxygen are colored in blue and red respectively. The prime symbol (‘) denotes residues of the opposite monomer.
(TIF)

S4 Fig Hydrogen bonding interactions and 2FoFc OMIT maps of Bd-NDPSase with ligands. Substrate carbons are shown in black, residue carbons are colored using the main chain color convention. Nitrogen and oxygen are colored in blue and red respectively. The prime symbol (‘) denotes residues of the opposite monomer. Hydrogen bonds are shown as orange dashes (top row), 2FoFc OMIT maps at σ = 1 are shown as a gray mesh around (1.6 Å) the ligands (middle and bottom rows). **Left column; A, D, G)** Wild type Bd-NDPSase in complex with glycerol (PDB ID 5C7Q). **Middle column; B, E, H)** E140Q Bd-NDPSase in complex with ADPR (PDB ID 5C7T)**. Right column; C, F, I)** E140Q Bd-NDPSase in complex with glucose (PDB ID 5C8L).
(TIF)

S5 Fig Structure of three domain-swapped dimeric Nudix sugar hydrolases. Ribbon representation in which one monomer is colored in a lighter shade. **A)** Bd-NDPSase (PDB ID 5C7T). **B)** Ec-NDPSase (PDB ID 3O61). **C)** Ec-ADPRase (PDBID 1KHZ). **D)** Structural alignment of the three Nudix sugar hydrolases.
(TIF)
