# Supplementary figures and images for: Structural and Enzymatic Characterization of a Nucleoside Diphosphate Sugar Hydrolase from Bdellovibrio bacteriovorus
Source: PLoS One. 2015 Nov 2;10(11):e0141716. doi: 10.1371/journal.pone.0141716 (PMC4629899; doi:10.1371/journal.pone.0141716)

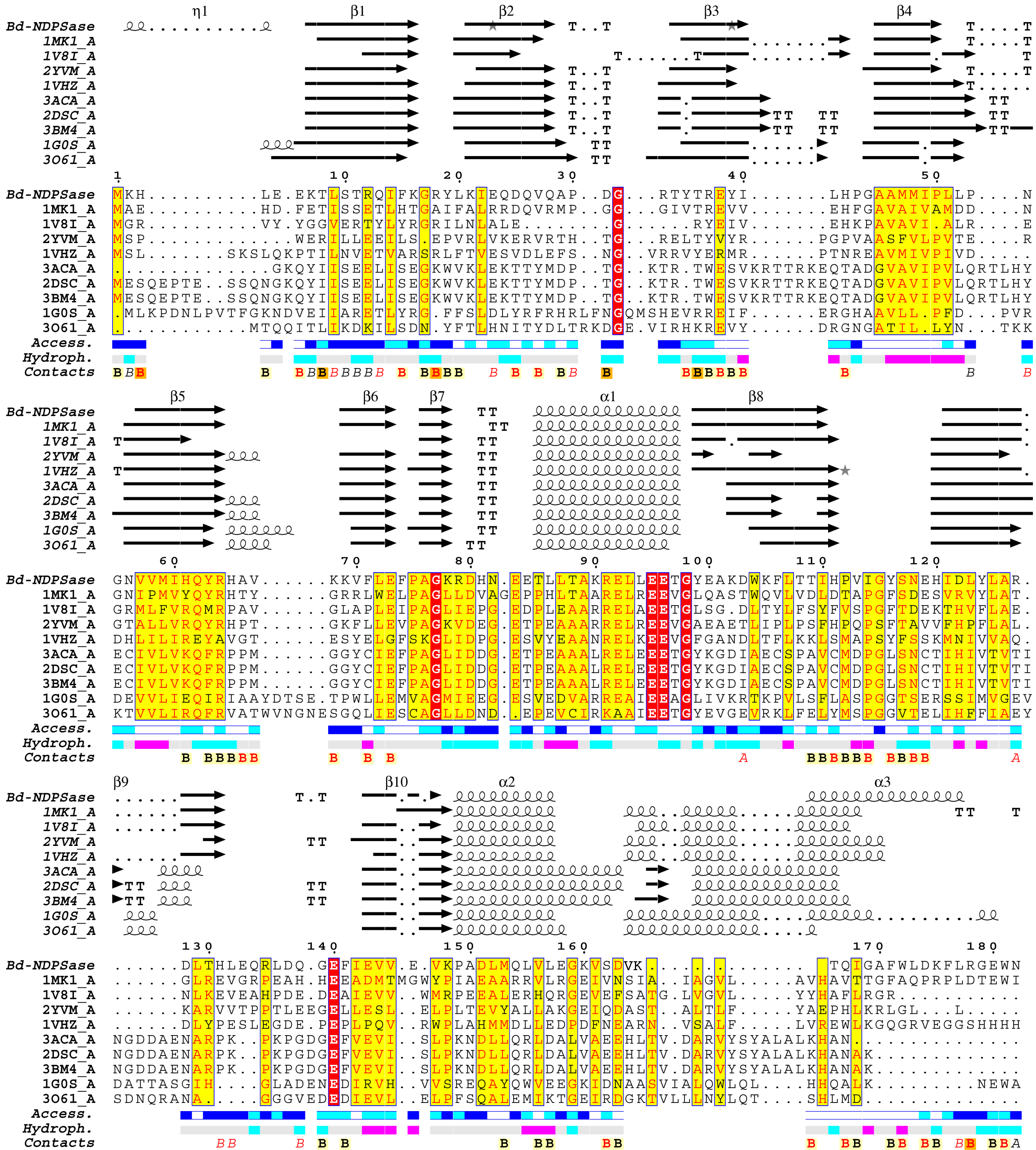

Supplement: S1 Fig — Chain A of the PDB entries 1MK1, 1V8I, 2YVM, 1VHZ, 3ACA, 2DSC, 3BM4, 1GOS, and 3O61 were aligned to chain A of Bd-NDPSase using the ENDscript web server. Accessibility: white is buried (A < 0.1), cyan is intermediate (0.1 ≤ A ≤ 0.4), blue is accessible (0.4 < A ≤ 1). Hydropathy: pink is hydrophobic (H>1.5), gray is intermediate (-1.5 ≤ H ≤ 1.5), and cyan is hydrophilic (H < -1.5). Contacts: yellow background is a non-crystallographic contact, orange background is both a crystallographic and a non-crystallographic contact, a red letter is a short range contact (ℓ < 3.2 Å), a black letter is a long range contact (3.2 Å ≤ ℓ ≤ 5.0 Å), an asterisk is a ligand contact, A and B denote the interacting chain. (TIF) [file pone.0141716.s001.tif]

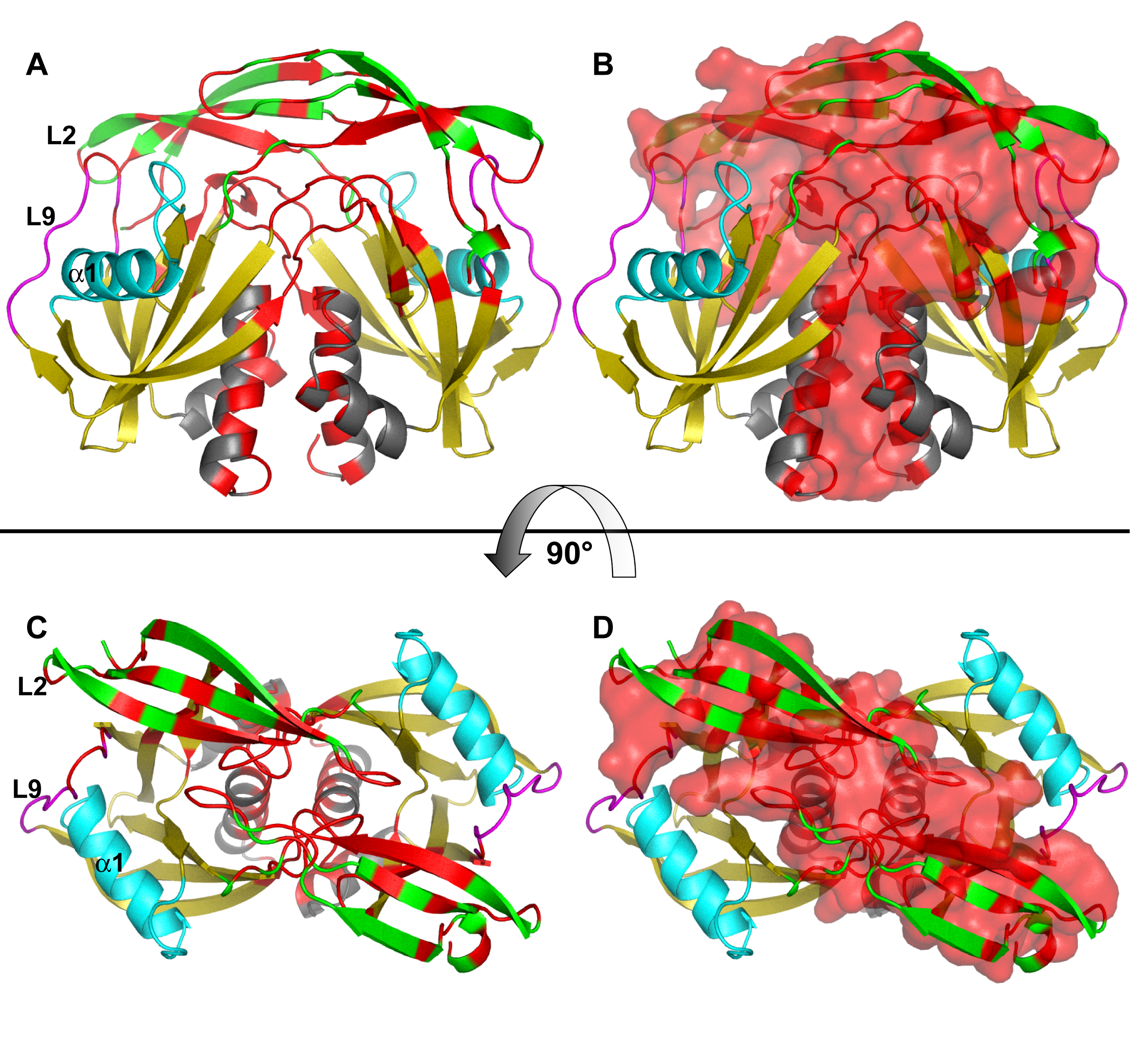

Supplement: S2 Fig — The dimer interface was calculated using the web server PISA. Structural elements are colored as in Figs 2–5. The side chains involved in dimer contacts are represented by red ribbons (A and C) and a red surface (B and D). Panels C-D are viewed at a 90° rotation from A-B. (TIF) [file pone.0141716.s002.tif]

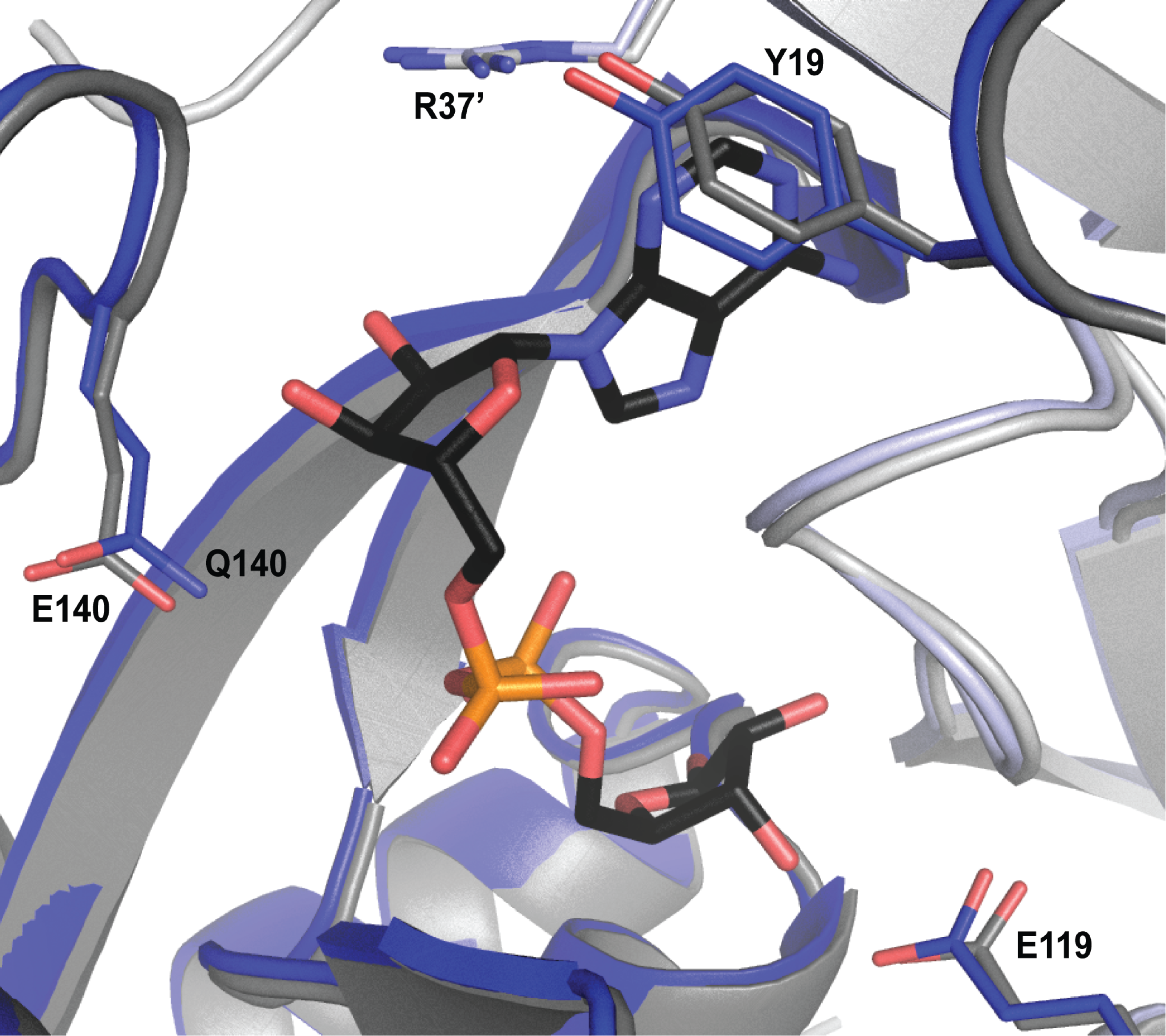

Supplement: S3 Fig — Ribbon representation in which wild type Bd-NDPSase bound to glycerol (PDB ID_5C7Q) is shown in gray and E140Q Bd-NDPSase bound to ADPR (PDB ID 5C7T) is shown in blue. One chain of the dimer is shown in a lighter shade. Substrate carbons are shown in black, residue carbons are colored using the main chain color convention. Nitrogen and oxygen are colored in blue and red respectively. The prime symbol (‘) denotes residues of the opposite monomer. (TIF) [file pone.0141716.s003.tif]

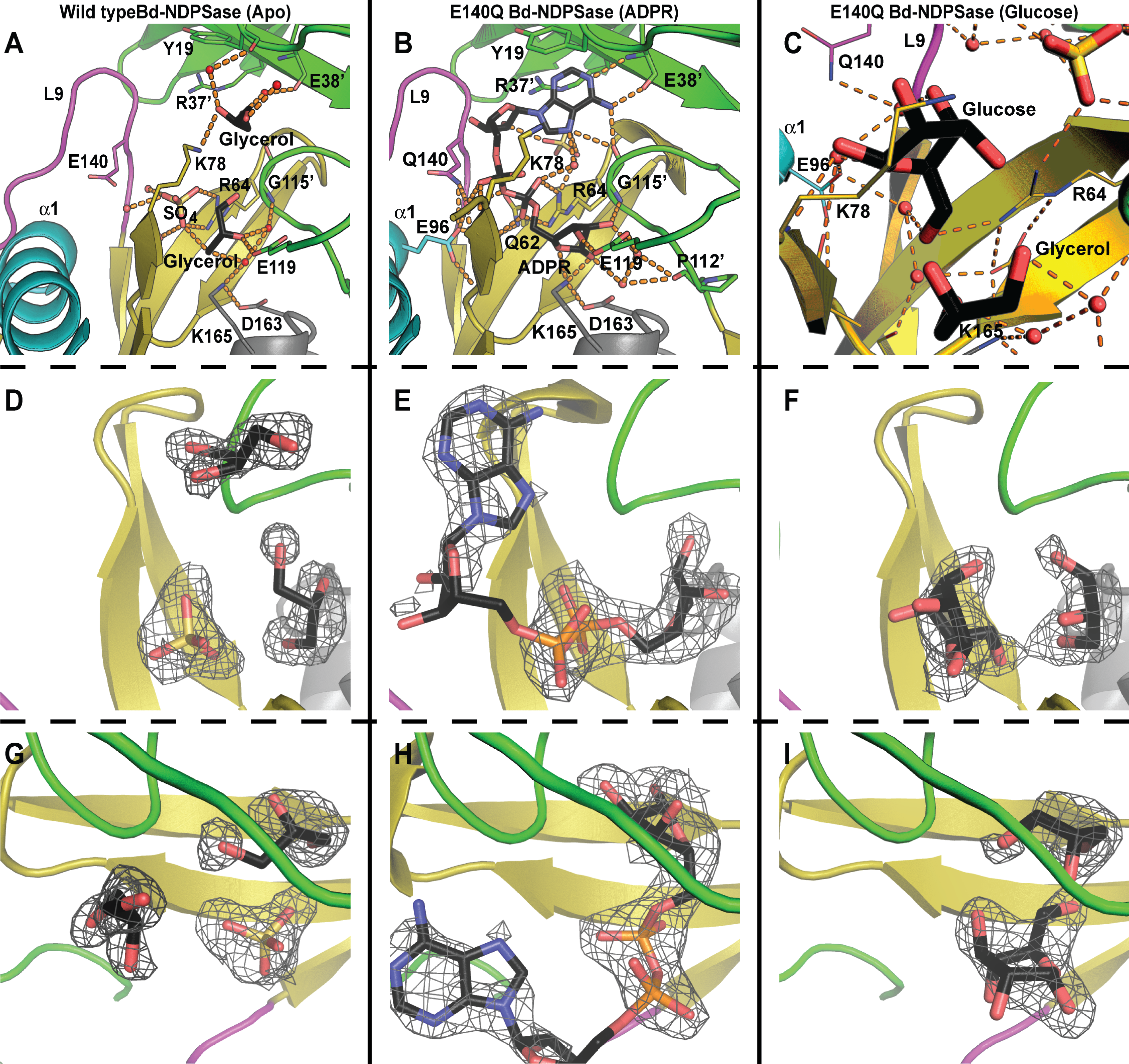

Supplement: S4 Fig — Substrate carbons are shown in black, residue carbons are colored using the main chain color convention. Nitrogen and oxygen are colored in blue and red respectively. The prime symbol (‘) denotes residues of the opposite monomer. Hydrogen bonds are shown as orange dashes (top row), 2FoFc OMIT maps at σ = 1 are shown as a gray mesh around (1.6 Å) the ligands (middle and bottom rows). Left column; A, D, G) Wild type Bd-NDPSase in complex with glycerol (PDB ID 5C7Q). Middle column; B, E, H) E140Q Bd-NDPSase in complex with ADPR (PDB ID 5C7T). Right column; C, F, I) E140Q Bd-NDPSase in complex with glucose (PDB ID 5C8L). (TIF) [file pone.0141716.s004.tif]

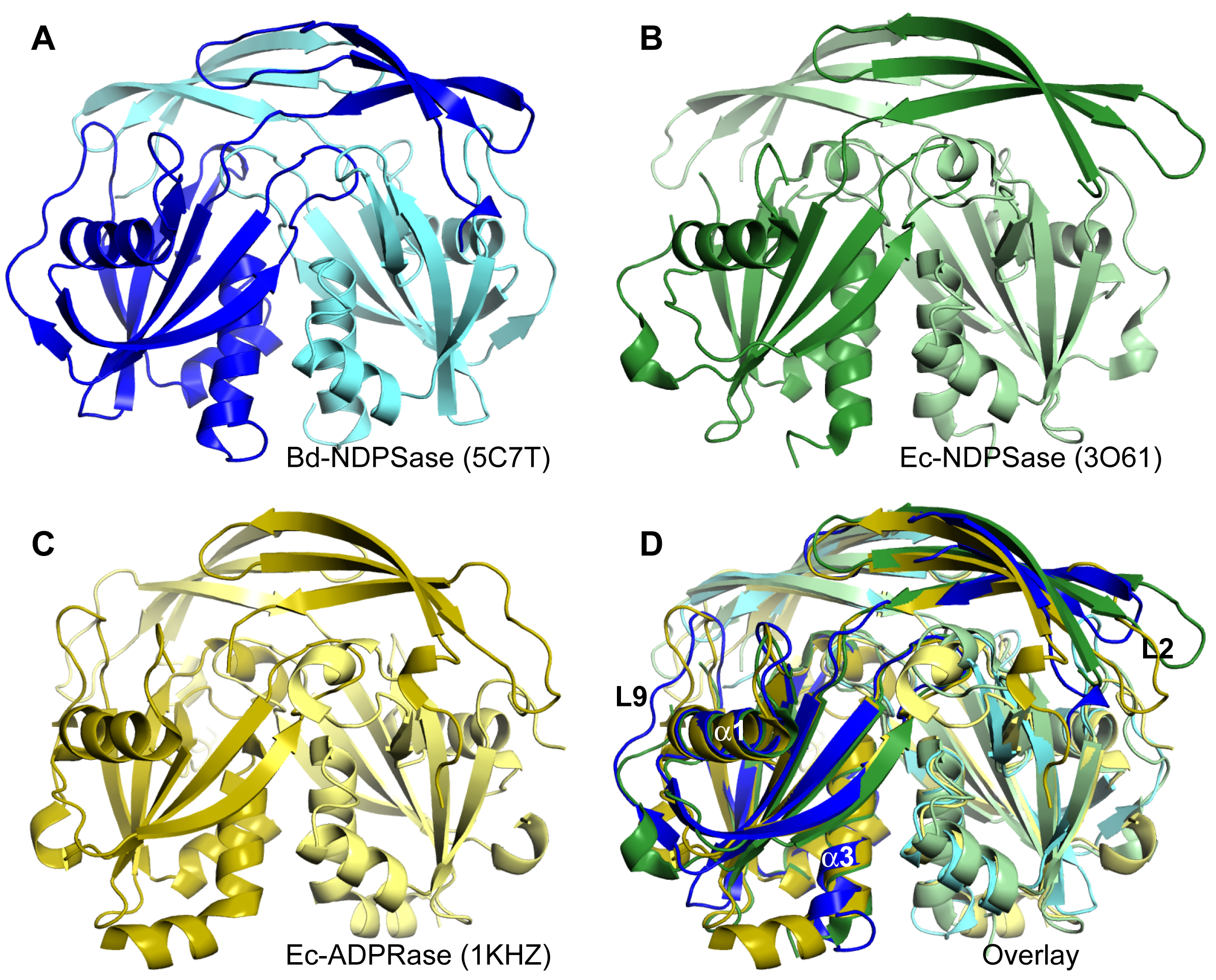

Supplement: S5 Fig — Ribbon representation in which one monomer is colored in a lighter shade. A) Bd-NDPSase (PDB ID 5C7T). B) Ec-NDPSase (PDB ID 3O61). C) Ec-ADPRase (PDBID 1KHZ). D) Structural alignment of the three Nudix sugar hydrolases. (TIF) [file pone.0141716.s005.tif]
